# Supplementary material for: Indefinitely stable iron(IV) cage complexes formed in water by air oxidation
Source: Nat Commun. 2017 Jan 19;8:14099. doi: 10.1038/ncomms14099 (PMC5253674; doi:10.1038/ncomms14099)
Supplement: Supplementary Information — Supplementary Figures, Supplementary Tables, Supplementary Methods and Supplementary References [file ncomms14099-s1.pdf]

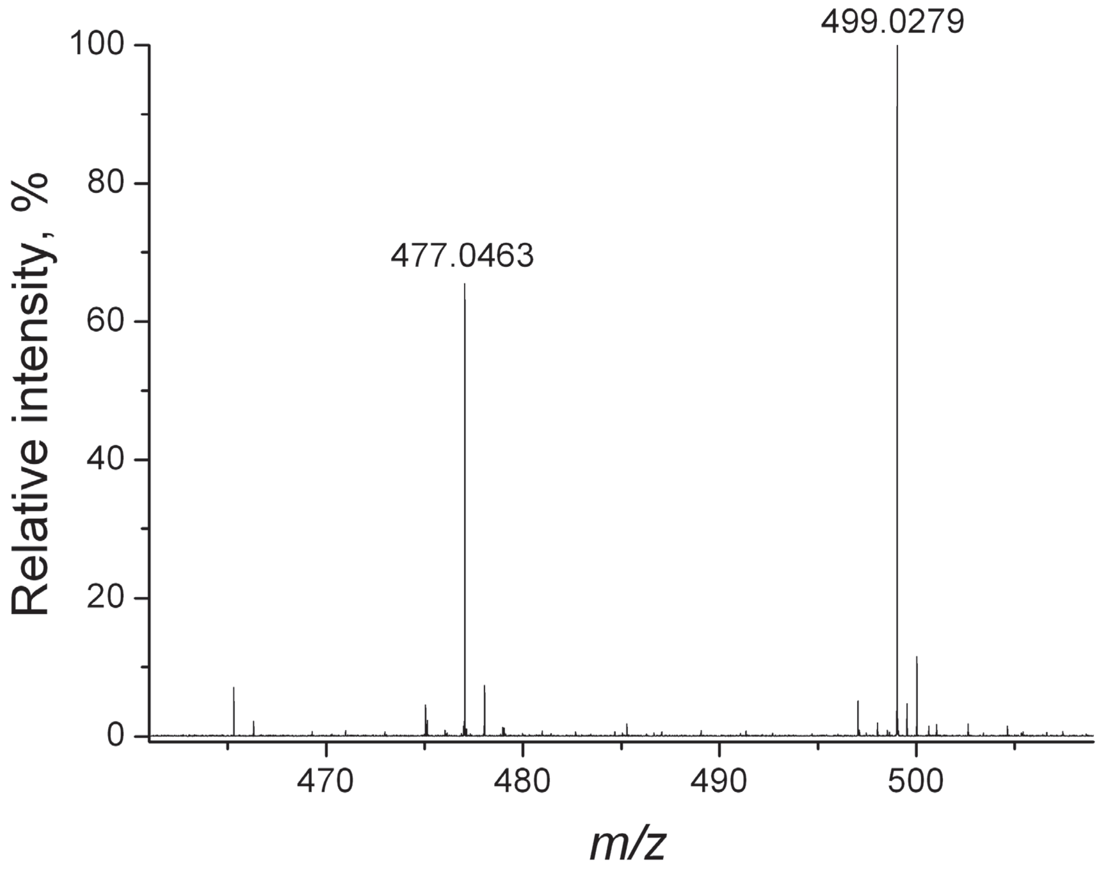

Supplementary Figure 1 | A fragment of ESI-MS spectrum (negative mode) of 4 in water-methanol (1:9) mixture. I, relative abundance (%); *m*/*z*, mass to charge ratio.

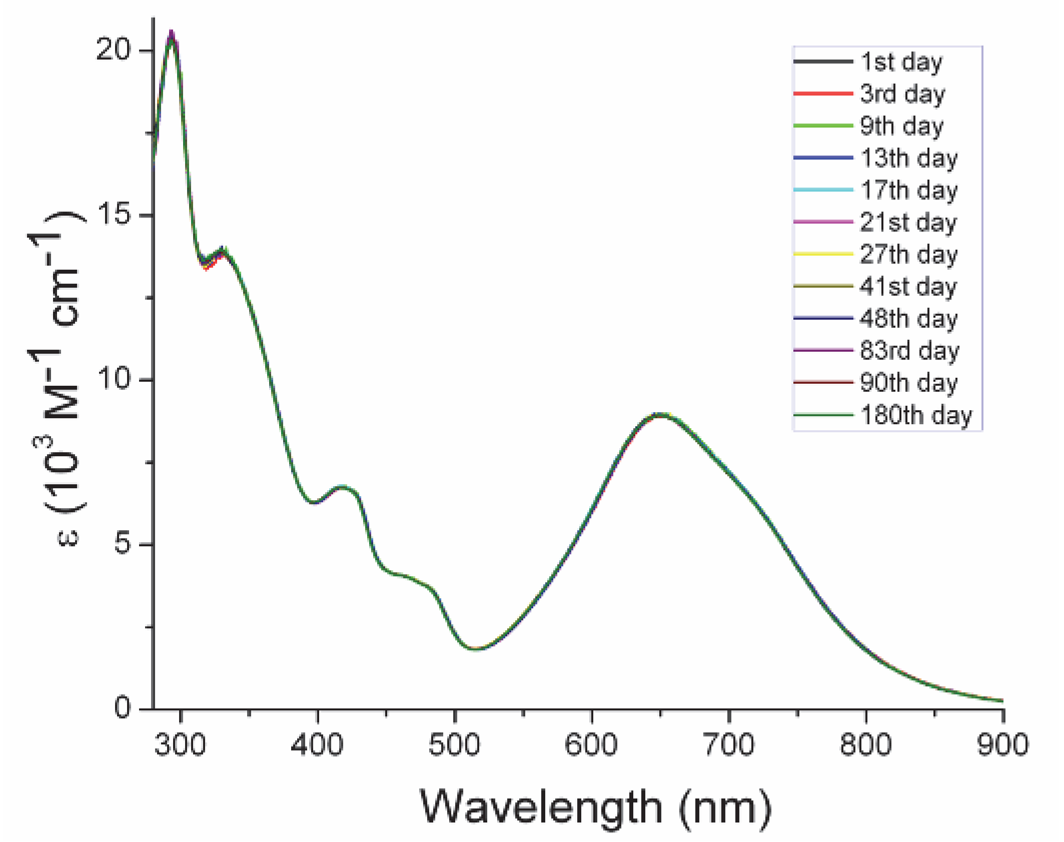

a

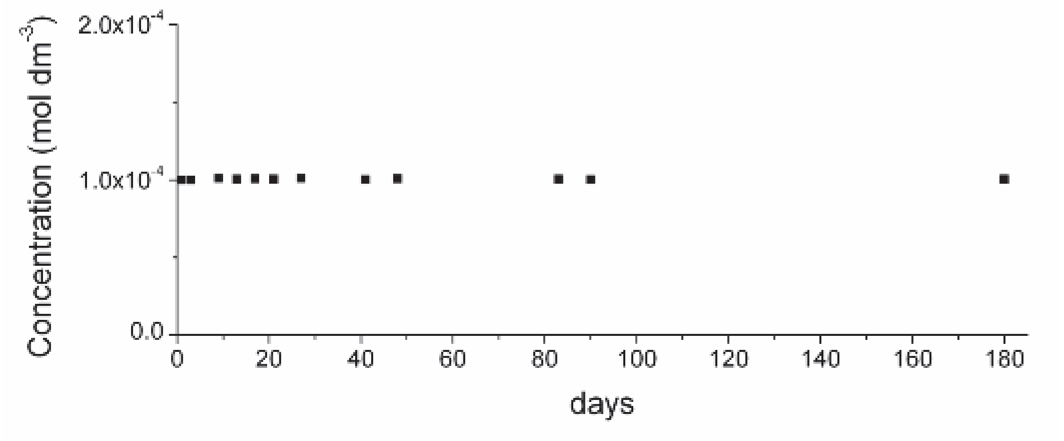

b

Supplementary Figure 2 | Stability of 3 in aqueous solution.(**a**) Electronic absorption spectra of 10^{-4} M aqueous solution measured during six months period at 20 ^{o}C. (**b**) Changes in concentration of **3** in aqueous solution (10^{-4} M) with time.

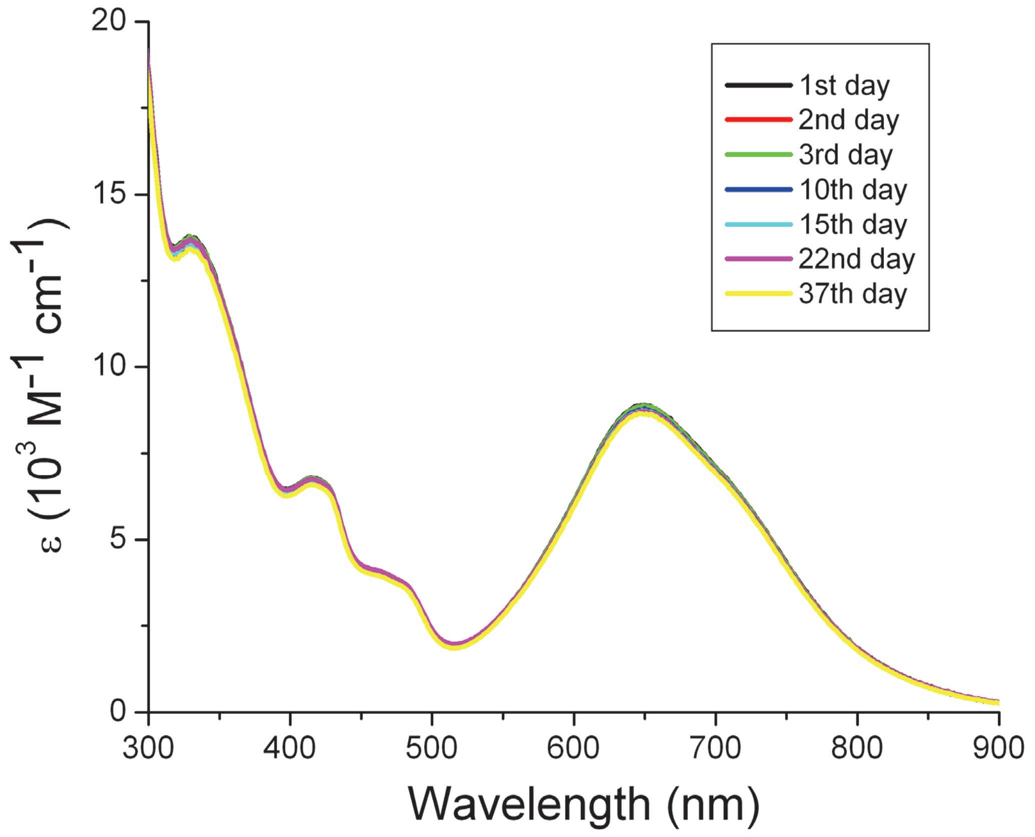

a

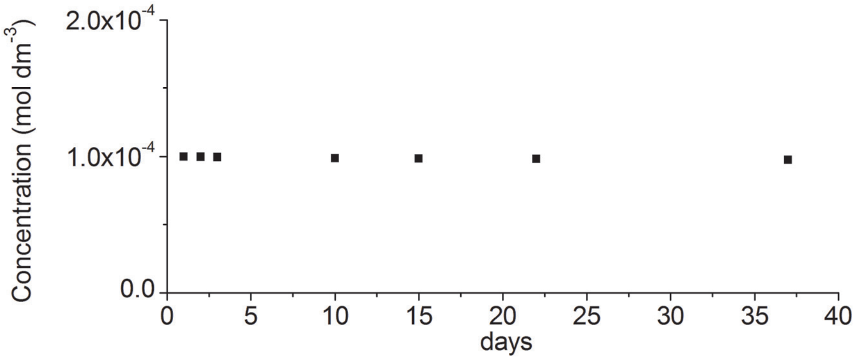

b

Supplementary Figure 3 | Stability of 3 in 0.1 M HCl. (**a**) Electronic absorption spectra of 10^{-4} M aqueous solution of **3** in 0.1 M HCl measured during one month period at 20 ^{o}C. The summary intensity decay on the 37th day was less than 3%. (**b**) Changes in concentration of **3** in aqueous solution (10^{-4} M) in 0.1 M HCl with time.

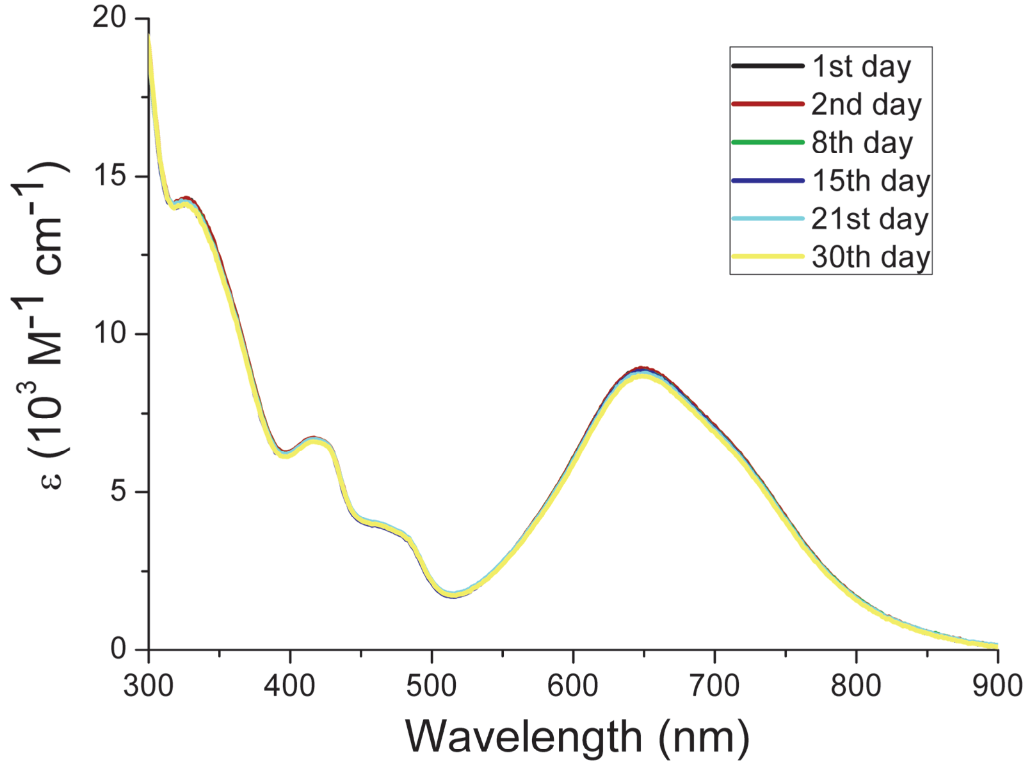

a

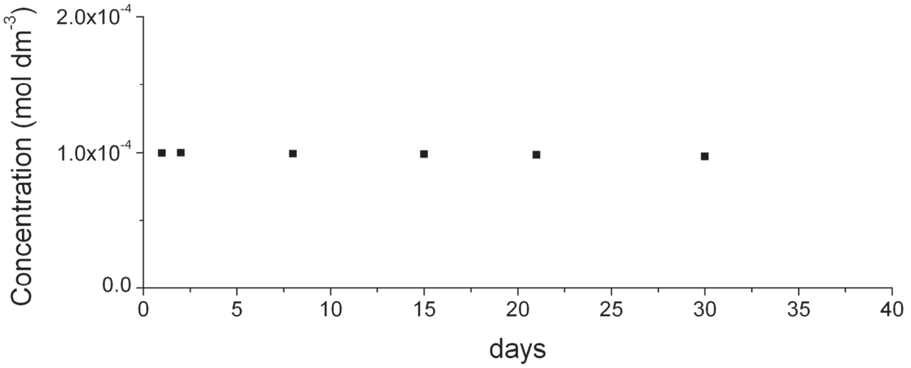

b

Supplementary Figure 4 | Stability of 3 in 0.1 M NaOH. (**a)** Electronic absorption spectra of 10^{-4} M aqueous solution of **3** in 0.1 M NaOH measured during one month period at 20 ^{o}C. The summary intensity decay on the 30th day was less than 3%. (**b**) Changes in concentration of **3** in aqueous solution (10^{-4} M) in 0.1 M NaOH with time.

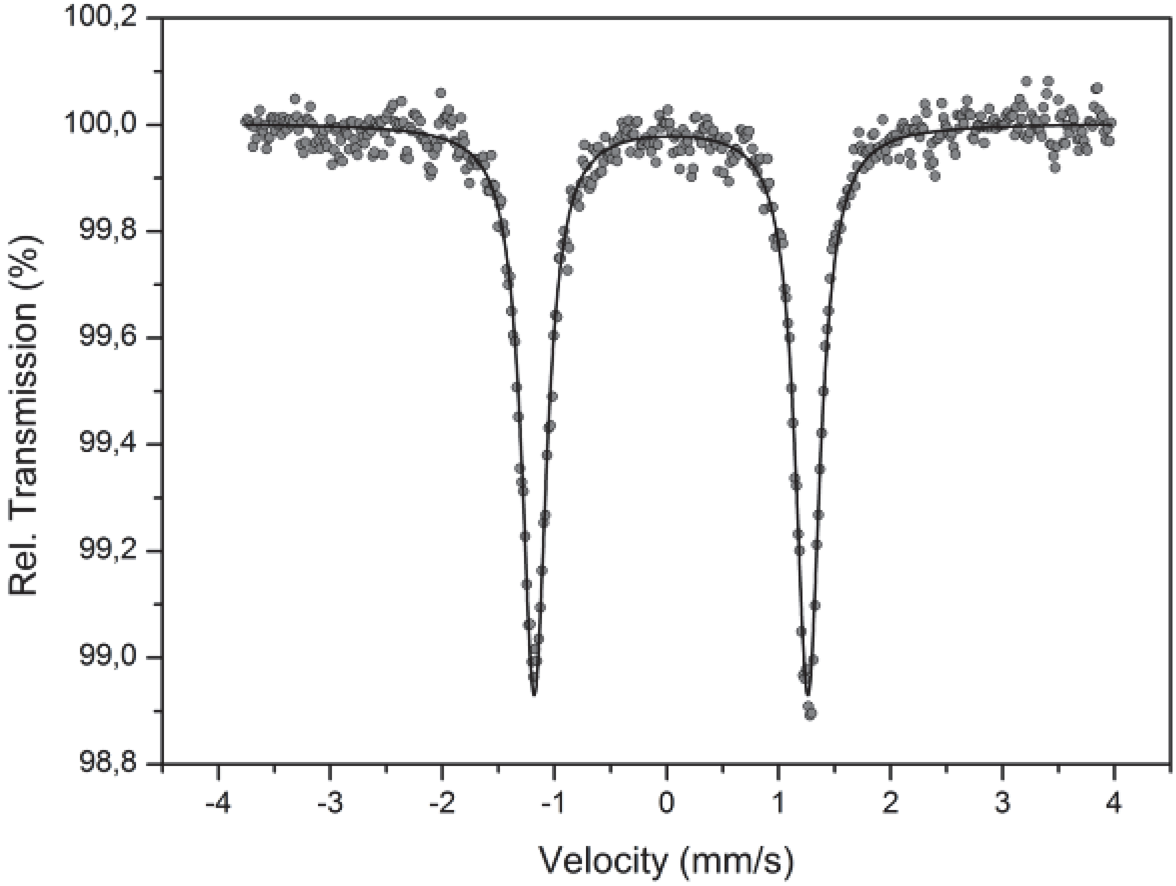

Supplementary Figure 5 | Zero-field ^{57}Fe Mössbauer spectrum of a microcrystalline sample of 3 recorded at 293 K in one year after isolation of the complex. The solid line represents the calculated Lorentzian doublet.

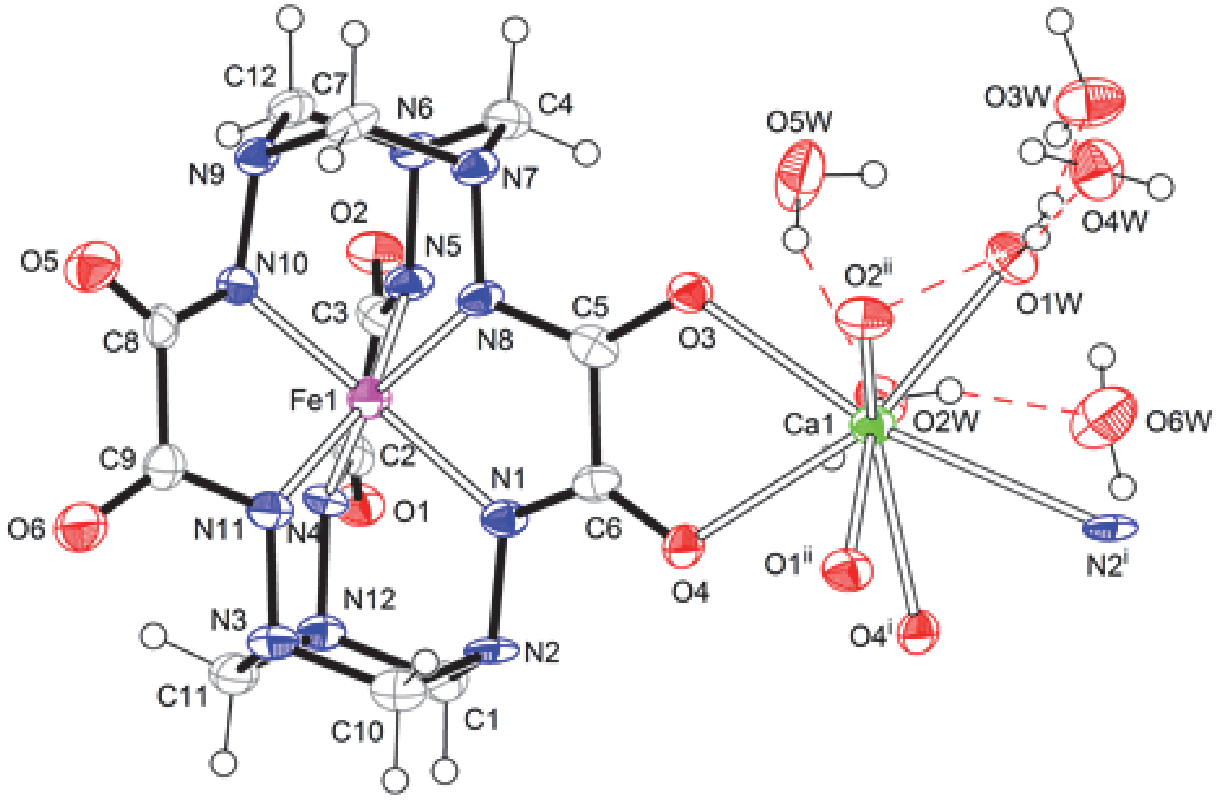

Supplementary Figure 6 | The molecular structure of Ca(Fe(L-6H))·6H_2O·*i*-PrOH (5). Displacement ellipsoids are drawn at the 50% probability level. Isopropanol solvate molecule was omitted for clarity.

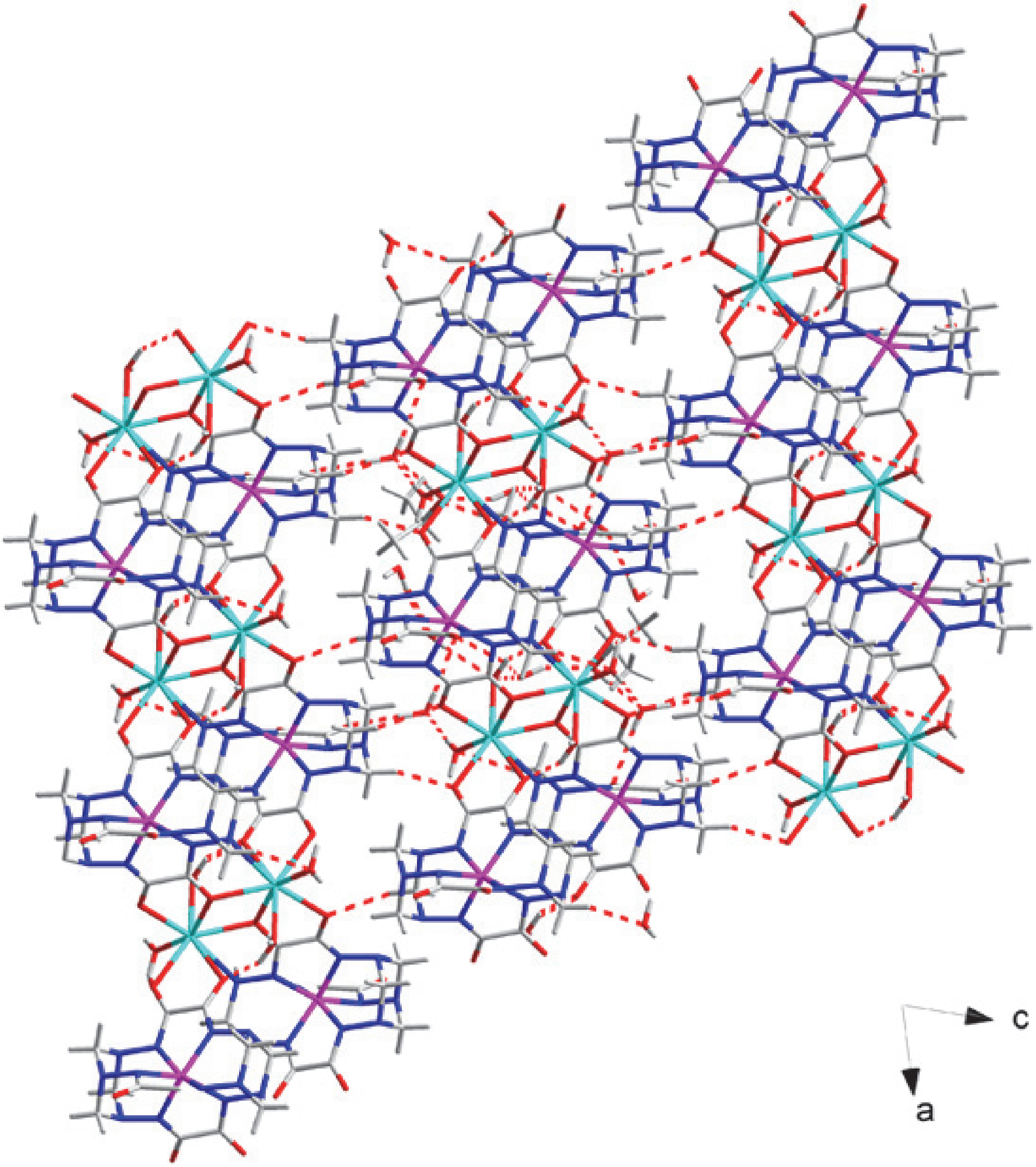

Supplementary Figure 7 | The crystal packing of Ca(Fe(L-6H))·6H_2O·*i*-PrOH (5).

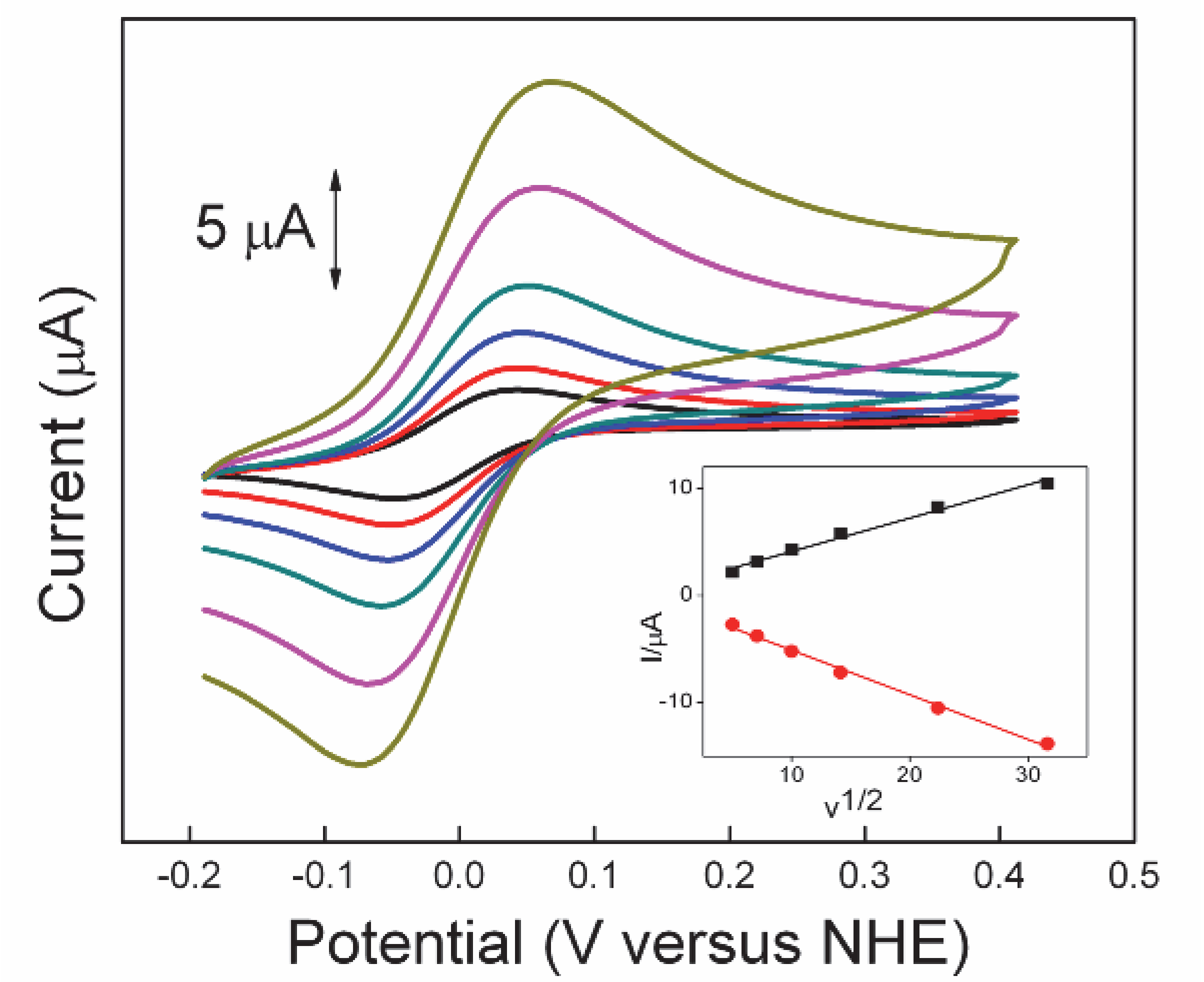

Supplementary Figure 8 | Cyclic voltammetric trace of 3 (1 mM) as a function of scan rate, recorded with $NaClO_4$ (0.1 M) as supporting electrolyte in aqueous solution. Scan rates (in mV s^{-1}): black – 25; red – 50; blue – 100; dark cyan – 200; magenta – 500; dark yellow – 1000. Inset: a plot of i_p as a function of the square root of the scan rate $v^{1/2}$, showing the linear relationship. For the numerical data and further details, see also Supplementary Table 9.

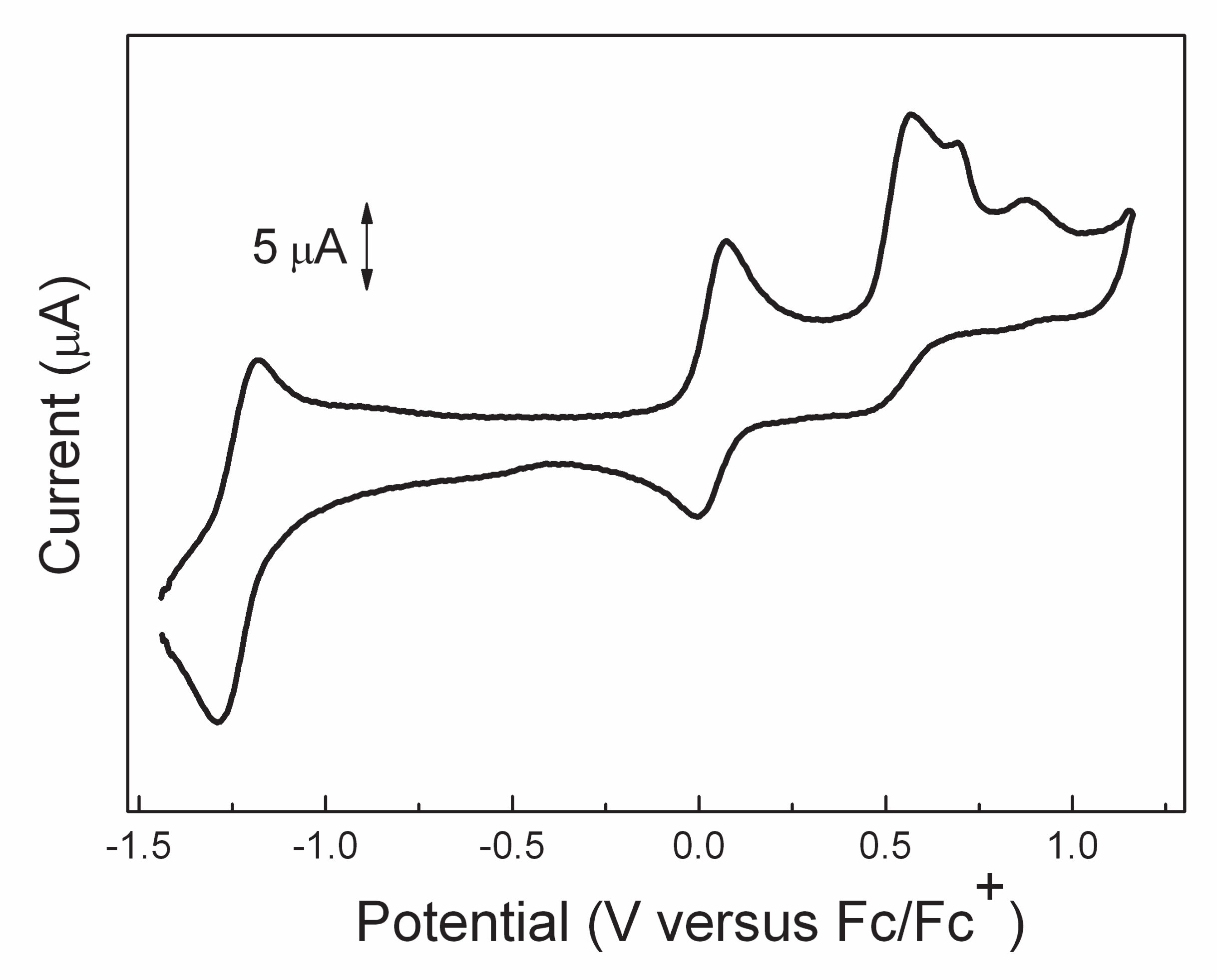

Supplementary Figure 9 | Cyclic voltammograms of 3 in acetonitrile solution at at a scan rate of 100 mV s^{-1}.

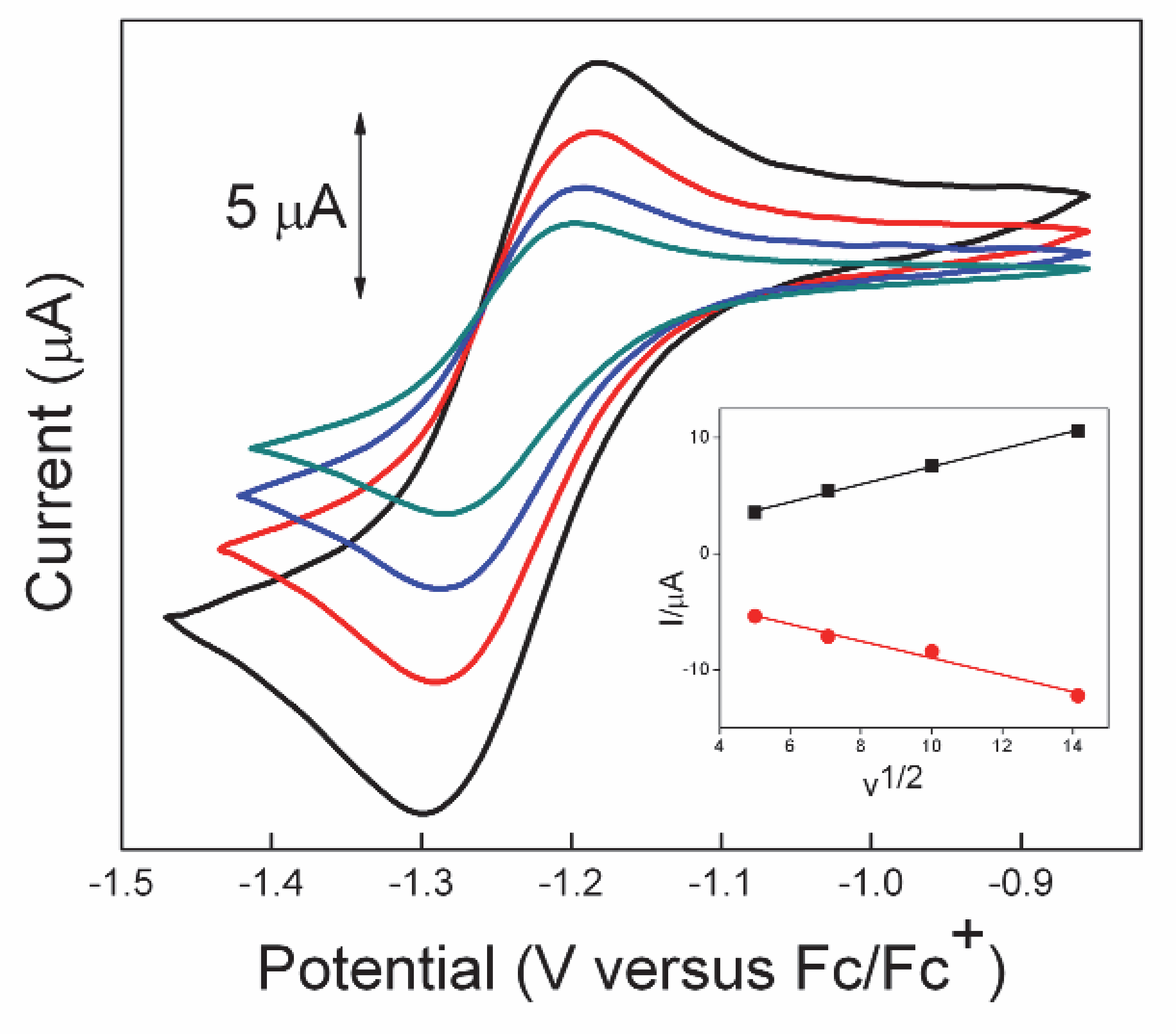

Supplementary Figure 10 | Cyclic voltammetric trace of 3 (1 mM) between -1.5 and -0.8 V vs. Fc/Fc$^+$ as a function of scan rate, recorded with Bu_4NClO_4 (0.1 M) as supporting electrolyte in acetonitrile solution. Scan rates (in mV s^{-1}): dark cyan – 25; blue – 50; red – 100; black – 200. Inset: a plot of i_p as a function of the square root of the scan rate $v^{1/2}$, showing the linear relationship. For the numerical data and further details, see also Supplementary Table 10.

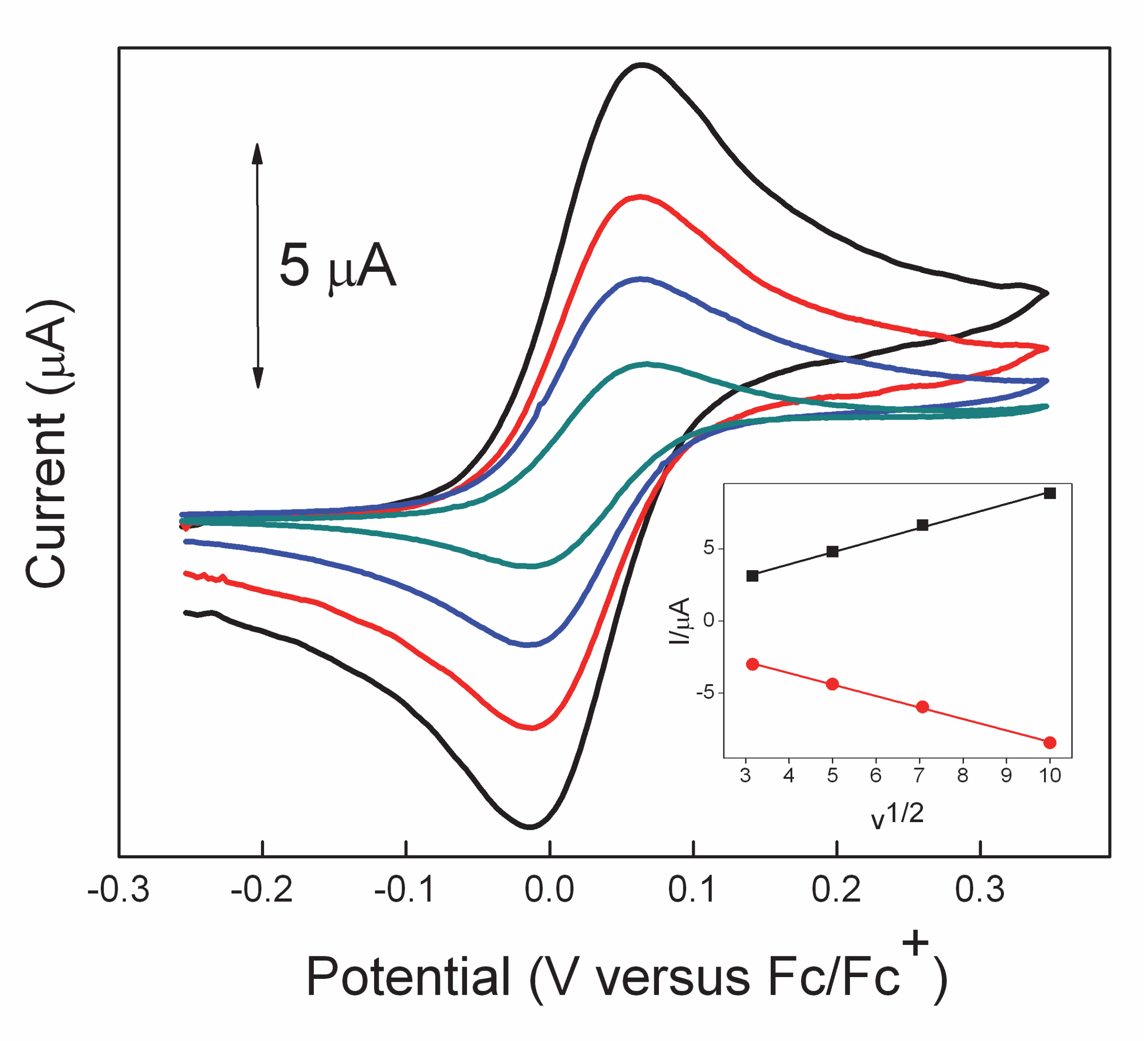

Supplementary Figure 11 | Cyclic voltammetric trace of 3 (1 mM) between -0.3 and +0.4 V vs. Fc/Fc$^+$ as a function of scan rate, recorded with Bu_4NClO_4 (0.1 M) as supporting electrolyte in acetonitrile solution. Scan rates (in mV s^{-1}): dark cyan – 10; blue – 25; red – 50; black – 100. Inset: a plot of i_p as a function of the square root of the scan rate $v^{1/2}$, showing the linear relationship. For the numerical data and further details, see also Supplementary Table 10.

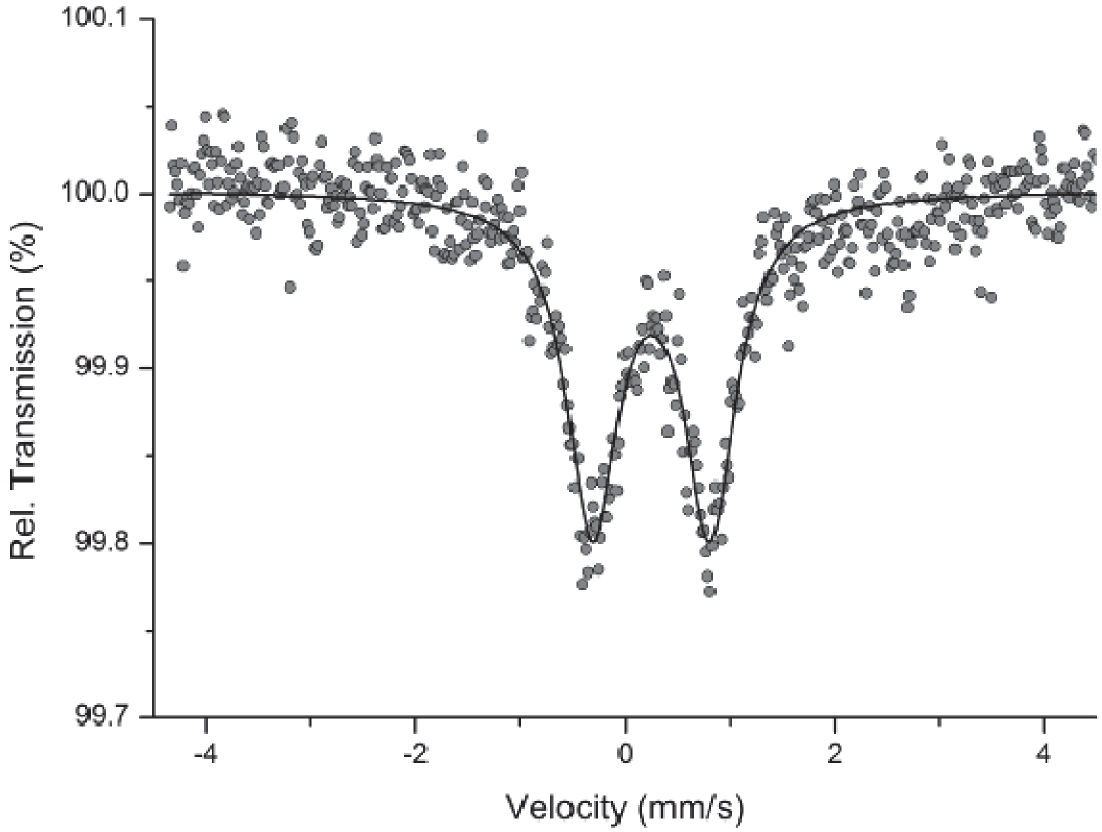

Supplementary Figure 12 | Zero-field ^{57}Fe Mössbauer spectrum of frozen aqueous solution of **3** reduced in the presence of excess of $Na_2S_2O_4$ recorded at 80 K. The solid line represents the calculated Lorentzian doublet. The main spectral parameters: $\delta = 0.25(3)$, $|\Delta E_Q| = 1.12(6)$ and $\Gamma_{FWHM} = 0.29(4)$ mm s^{-1}.

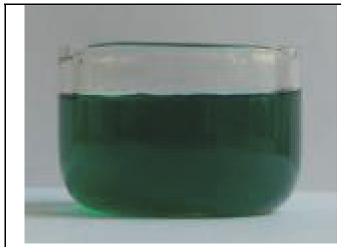	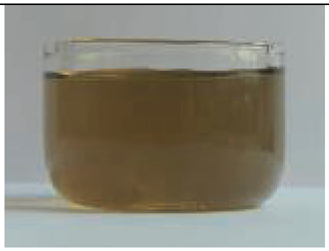	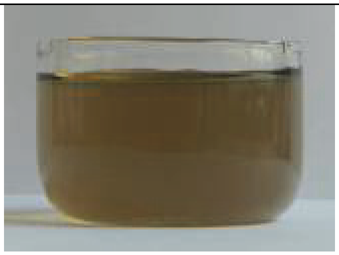	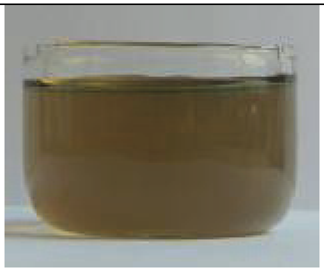	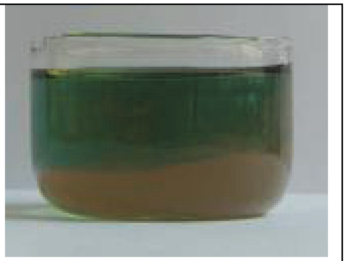
Starting solution	Immediately after adding of sodium dithionite	After 1 minute	After 5 minutes	After 30 minutes

Supplementary Figure 13 | Actual photographs of aqueous solution of 3 (10^{-4} M) before and after addition of crystalline sodium dithionite. The photographs gradual recovery of the initial green colour of the solution in the course of aerial oxidation without stirring at 20 ^{o}C. pH = 10.01 (0.1 M 2-(cyclohexylamino)ethanesulfonic acid (CHES) buffer).

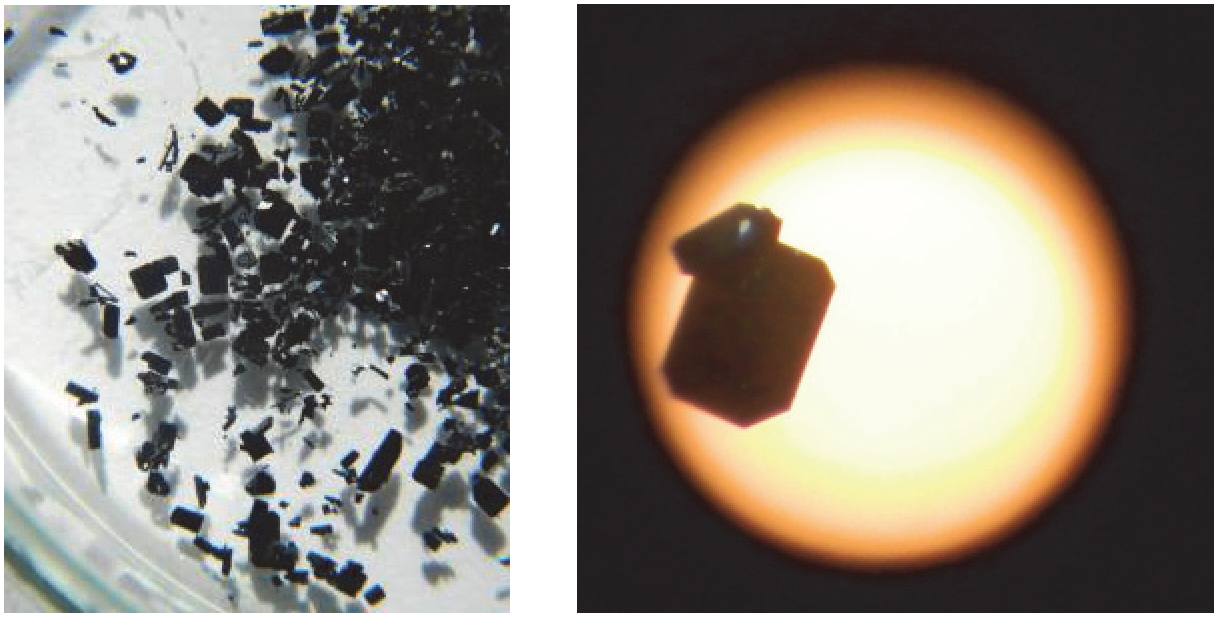

a b

Supplementary Figure 14 | Actual photographs of crystalline samples of **3**. (**a**) Bulk crystals. (**b**) A single crystal at 16x magnification.

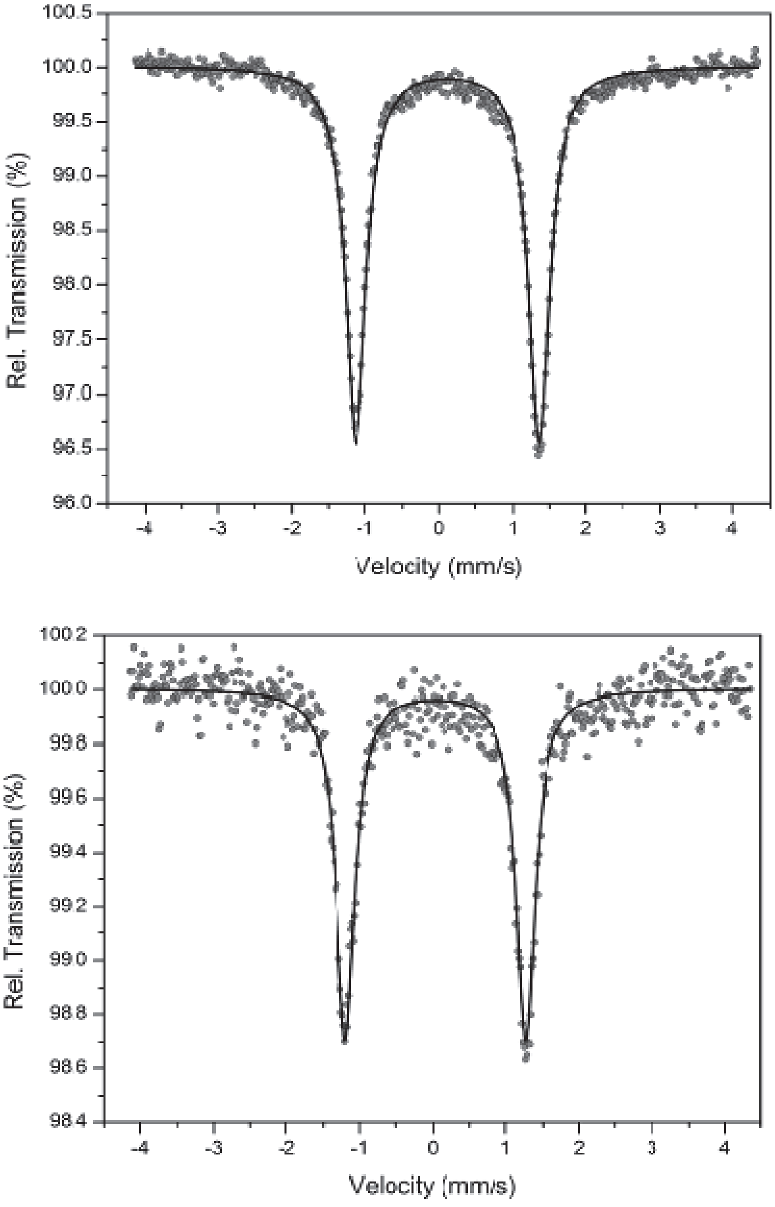

Supplementary Figure 15 | Zero-field ^{57}Fe Mössbauer spectra of a microcrystalline sample of 1. (**a**) Recorded at 80 K. (**b**) Recorded at 293 K. The solid lines represent the calculated Lorentzian doublets. The main spectral parameters: $\delta =$ 0.116(2), $|\Delta E_Q|$ = 2.495(4) and Γ_{FWHM} = 0.170(3) mm s^{-1} at 80 K and $\delta =$ 0.037(6), $|\Delta E_Q|$ = 2.471(10) and Γ_{FWHM} = 0.156(8) mm s^{-1} at 293 K. The isomer shift values are decreased by ca. 0.08 mm s^{-1} with increasing temperature from 80 to 293 K as a result of the second-order Doppler shift[1]. ΔE_Q was found to be independent at T<293K that implies a well-isolated orbital ground state.

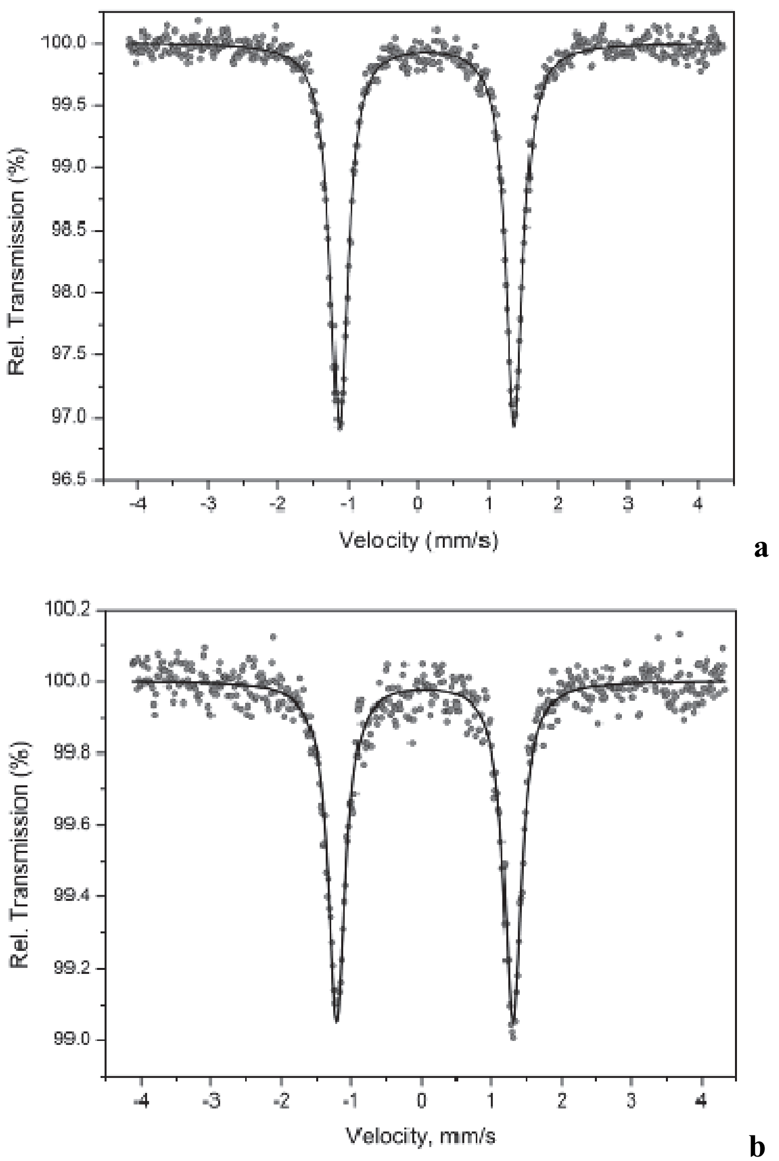

Supplementary Figure 16 | Zero-field ^{57}Fe Mössbauer spectra of a microcrystalline sample of 3. (**a**) Recorded at 80 K. (**b**) Recorded at 293 K. The solid lines represent the calculated Lorentzian doublets. The main spectral parameters: $\delta =$ 0.121(3), $|\Delta E_Q|$ = 2.505(5) and Γ_{FWHM} = 0.149(4) mm s^{-1} at 80 K and $\delta =$ 0.045(5), $|\Delta E_Q|$ = 2.511(10) and Γ_{FWHM} = 0.151(8) mm s^{-1} at 293 K. The isomer shift values are decreased by ca. 0.08 mm s^{-1} with increasing temperature from 80 to 293 K as a result of the second-order Doppler shift[1]. ΔE_Q was found to be independent at T<293K that implies a well-isolated orbital ground state.

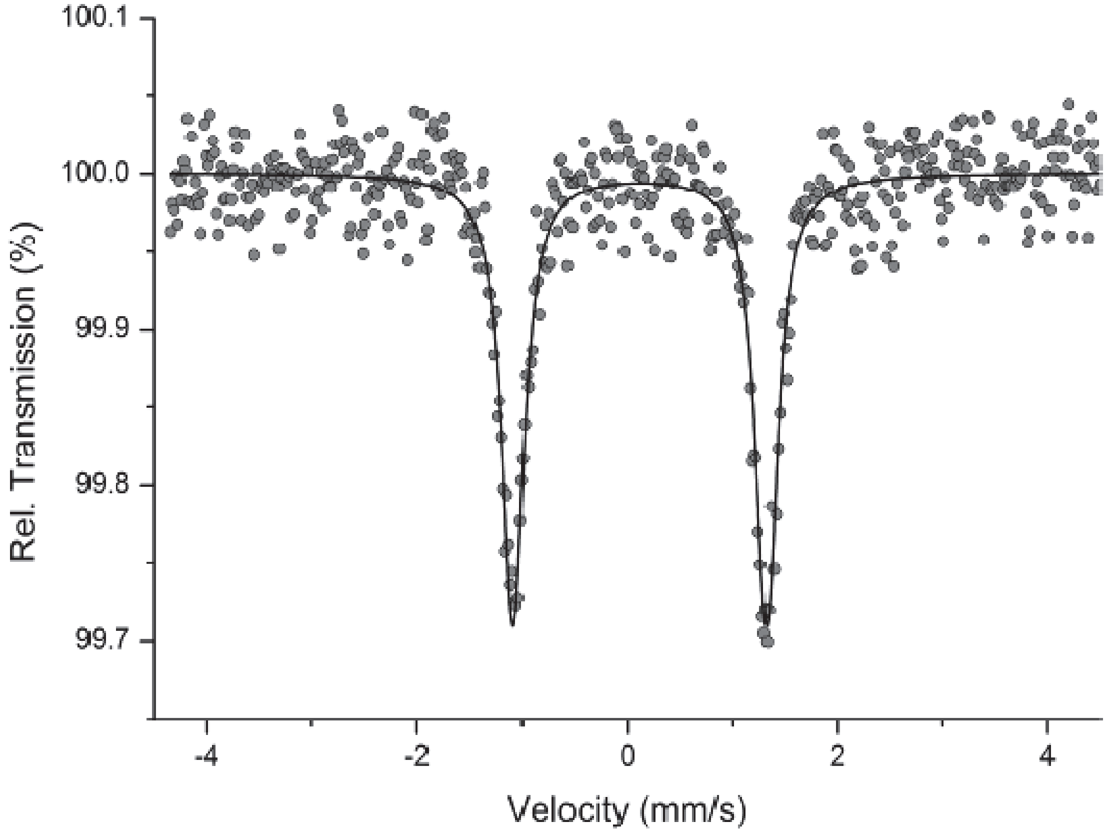

Supplementary Figure 17 | Zero-field ^{57}Fe Mössbauer spectrum of frozen aqueous solution of 3 recorded at 80 K. The solid line represents the calculated Lorentzian doublet. The main spectral parameters: $\delta =$ 0.12(1), $|\Delta E_Q|$ = 2.43(2) and Γ_{FWHM} = 0.13(1) mm s^{-1}.

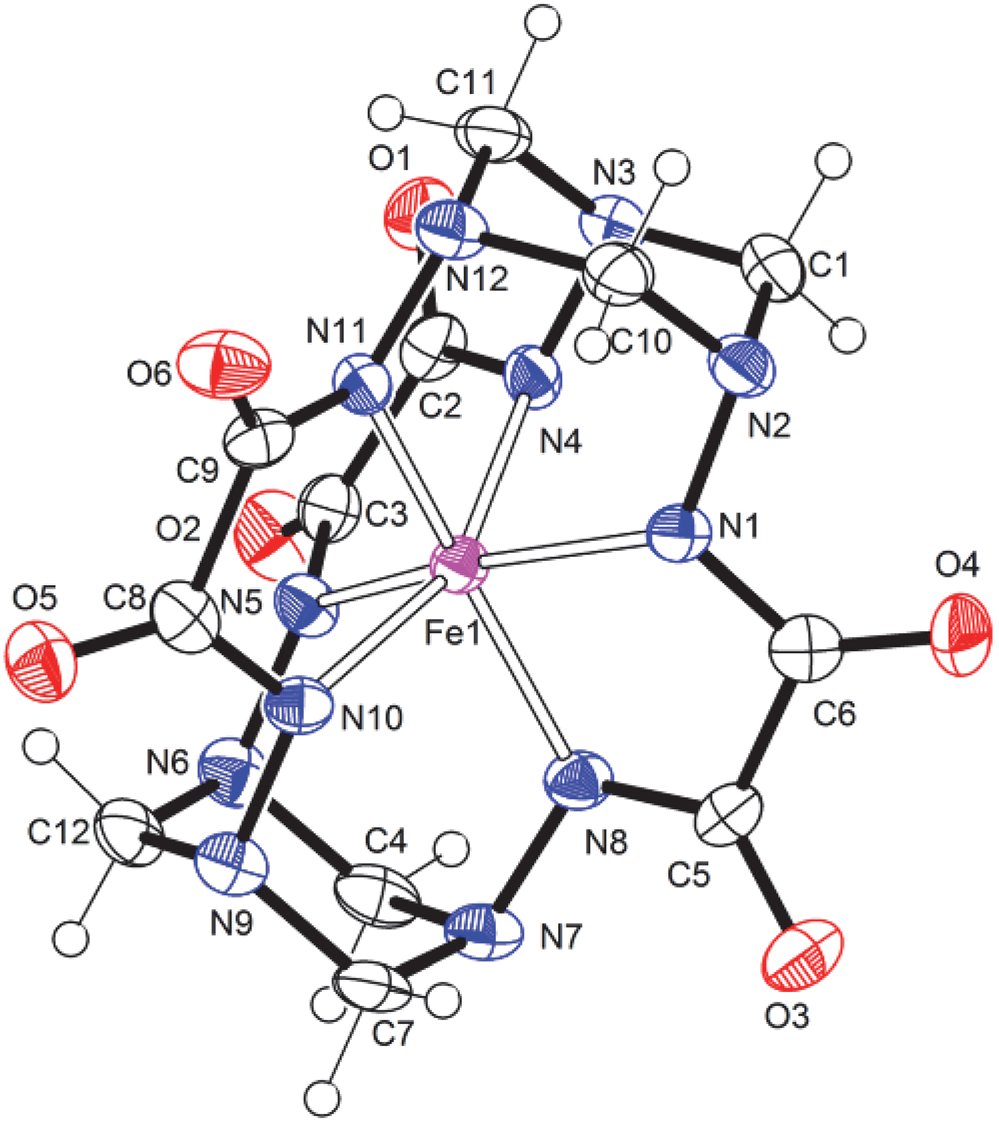

Supplementary Figure 18 | The molecular structure of complex anion in $(Bu_4N)_2$[Fe(L-6H)]·$7CHCl_3$ (2). Displacement ellipsoids are drawn at the 50% probability level.

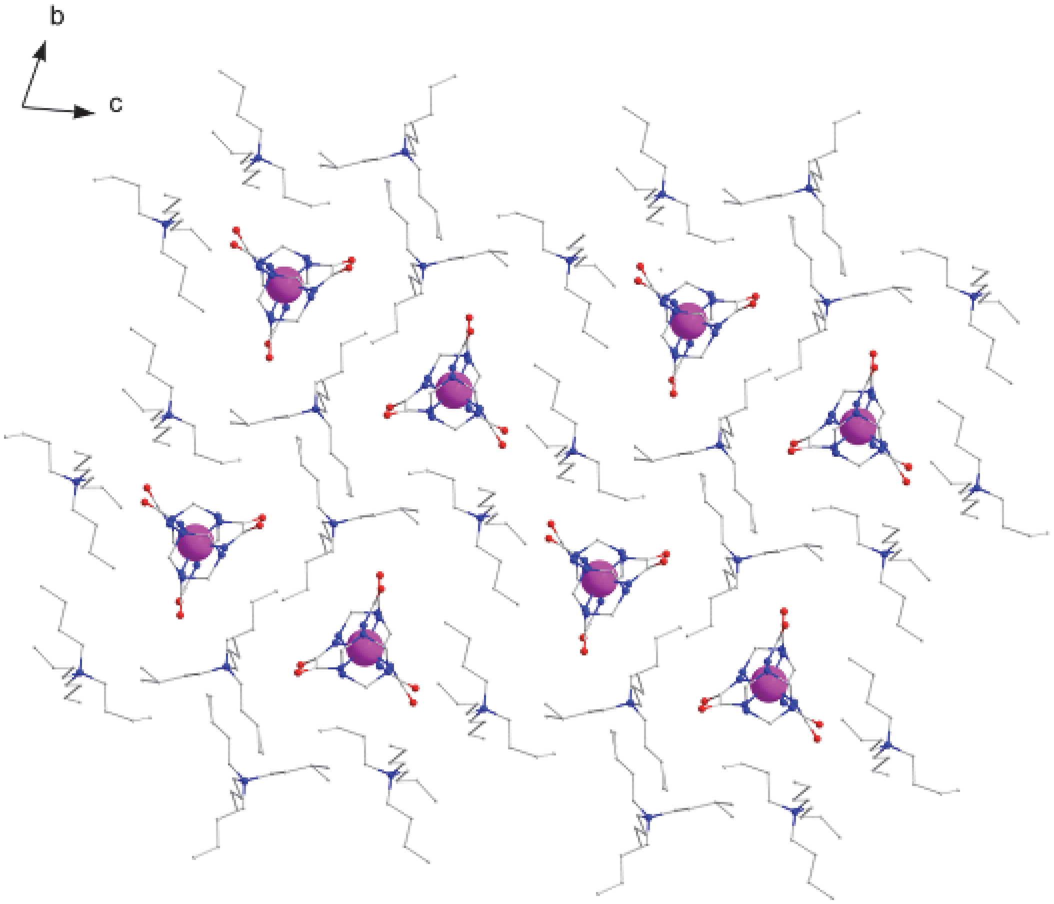

Supplementary Figure 19 | The crystal packing of $Bu_4N)_2[Fe(L\text{-}6H)]\cdot 7CHCl_3$ (2). Hydrogen atoms and chloroform molecules are omitted for clarity.

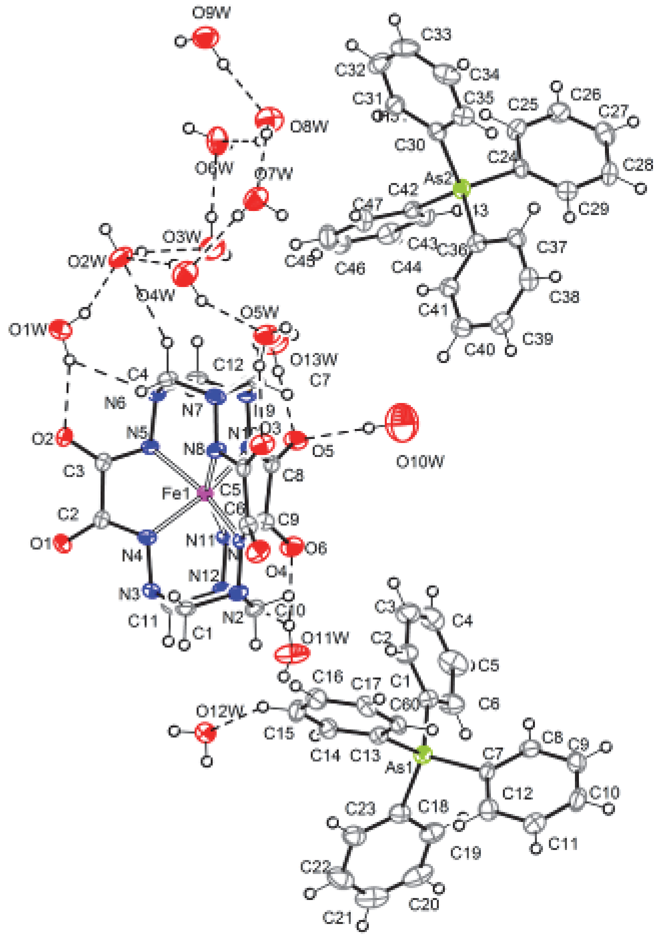

Supplementary Figure 20 | The molecular structure of $(Ph_4As)_2[Fe(L\text{-}6H)]\cdot 13H_2O$ (3). Displacement ellipsoids are drawn at the 50% probability level.

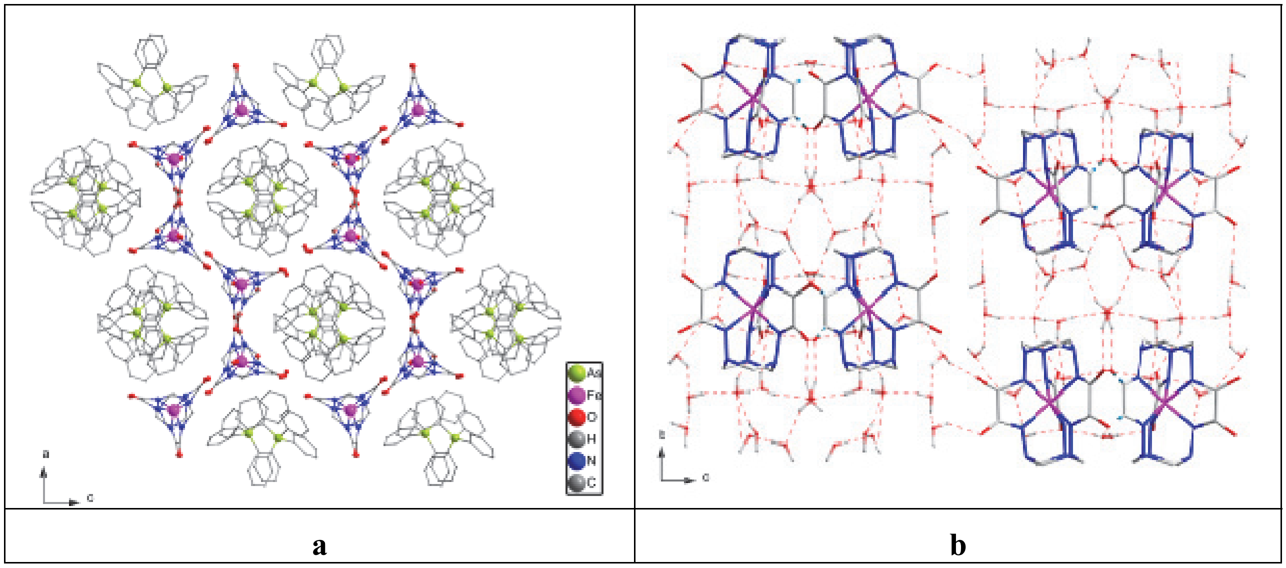

a	b

Supplementary Figure 21 | The crystal structure of $(Ph_4As)_2[Fe(L\text{-}6H)]\cdot 13H_2O$ (3). (**a**) The crystal packing (hydrogen atoms and water molecules are omitted for clarity). (**b**) The extensive system of H-bonds between clathrochelate anions and water molecules (Ph_4As^+ ions are omitted for the sake of clarity).

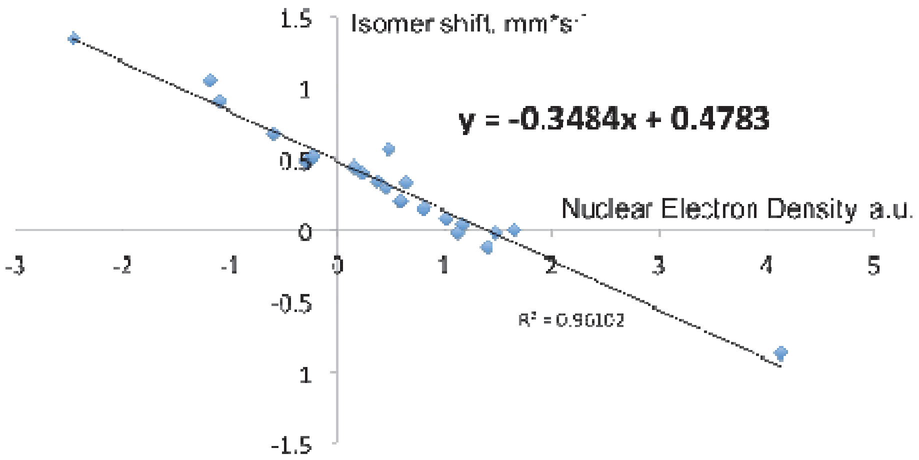

Supplementary Figure 22 | Calibration of B3LYP functional for the prediction of ^{57}Fe isomer shifts. The calibration procedure consists of calculating the electron density at the nuclei of interest and comparison to the experimentally known isomer shift values. The linear correspondence is then fitted to a straight line using the least squares method. Complexes used for this figure are listed below.

Supplementary Table 1 | The main geometrical parameters of the coordination sphere for **2**, **3** and **5**.

	**2**	**3**	**5**
φ, º	31.9	28.0	30.1
α, º	79.99	79.23	80.53
a, Å	1.954	1.957	1.948
h, Å	2.36	2.38	2.39

φ – a distortion angle (φ=0º for a trigonal prism and φ=60º for a trigonal antiprism)

α – the bite angle

a – the distance between the encapsulated metal ion and the coordinated nitrogen atom

h – the distance between the coordination polyhedron bases

Supplementary Table 2 | Selected bond distances (Å) and angles (°) for **2**, **3** and **5**.

	**2**	**3**	**5**
Fe1 – N1	1.955 (5)	1.968 (3)	1.961 (5)
Fe1 – N4	1.942 (5)	1.952 (3)	1.961 (5)
Fe1 – N5	1.947 (5)	1.969 (3)	1.938 (6)
Fe1 – N8	1.953 (5)	1.945 (3)	1.965 (5)
Fe1 – N10	1.961 (5)	1.950 (3)	1.946 (5)
Fe1 – N11	1.961 (5)	1.958 (3)	1.915 (5)
N1 – C6	1.341 (8)	1.347 (4)	1.309 (8)
N4 – C2	1.357 (7)	1.332 (4)	1.331 (8)
N5 – C3	1.337 (7)	1.330 (4)	1.341 (8)
N8 – C5	1.333 (8)	1.332 (4)	1.340 (8)
N10 – C8	1.337 (8)	1.336 (4)	1.356 (8)
N11 – C9	1.341 (8)	1.338 (4)	1.351 (7)
N1 – N2	1.417 (6)	1.418 (4)	1.436 (6)
N3 – N4	1.417 (6)	1.425 (4)	1.427 (6)
N5 – N6	1.428 (6)	1.424 (4)	1.432 (6)
N7 – N8	1.427 (6)	1.432 (4)	1.415 (6)
N9 – N10	1.426 (6)	1.419 (4)	1.403 (6)
N11 – N12	1.412 (6)	1.421 (4)	1.427 (6)
O1 – C2	1.216 (7)	1.238 (4)	1.249 (7)
O2 – C3	1.236 (7)	1.237 (4)	1.228 (8)
O3 – C5	1.232 (7)	1.234 (4)	1.251 (7)
O4 – C6	1.221 (7)	1.236 (4)	1.257 (7)
O5 – C8	1.221 (7)	1.241 (4)	1.231 (7)
O6 – C9	1.222 (7)	1.232 (4)	1.237 (7)
N1 – Fe1 – N4	87.42 (19)	86.76 (12)	85.0 (2)
N1 – Fe1 – N8	79.86 (19)	79.46 (12)	80.5 (2)
N1 – Fe1 – N11	87.25 (19)	86.65 (12)	86.1 (2)
N4 – Fe1 – N5	80.0 (2)	78.84 (12)	80.3 (2)
N4 – Fe1 – N11	86.5 (2)	87.47 (12)	86.7 (2)
N5 – Fe1 – N8	87.5 (2)	85.98 (12)	85.9 (2)
N5 – Fe1 – N10	87.2 (2)	87.42 (12)	87.3 (2)
N8 – Fe1 – N10	87.7 (2)	86.87 (13)	87.1 (2)
N10 – Fe1 – N11	80.05 (19)	79.39 (12)	80.8 (2)

Supplementary Table 3 | Comparison of the bond lengths for the BP86 optimized geometry (DFT) and crystallographically determined structure of complex anion of **3** (X-ray). All bond lengths are in Å. The numbering of the nitrogen atoms corresponds to Fig. 1b of the main text.

	X-ray	DFT
Fe1–N1	1.968(3)	1.960
Fe1–N4	1.952(3)	1.958
Fe1–N5	1.969(3)	1.959
Fe1–N8	1.946(3)	1.958
Fe1–N10	1.949(3)	1.958
Fe1–N11	1.957(3)	1.959

Supplementary Table 4 | The voltammetric data for complexes **1** and **3** (10^{-3} M) in aqueous solution (0.1 M $NaClO_4$ as supporting electrolyte) at 298 K.

Compound	r ($mV\ s^{-1}$)	$E_{p,a}$ (V) vs. Ag/AgCl	$E_{p,c}$ (V) vs. Ag/AgCl	ΔE_p(V)	$i_{p,a}$ (μA)	$i_{p,c}$ (μA)	$i_{p,a}/i_{p,c}$	**$E_{1/2}$ (V) vs. Ag/AgCl**	**$E_{1/2}$ (V) vs. NHE**
**1**	10	-0.200	-0.293	0.093	0.647	0.377	1.9	**-0.247**	**-0.036**
**1**	25	-0.195	-0.296	0.101	0.538	0.895	0.6	**-0.246**	**-0.035**
**1**	50	-0.197	-0.302	0.105	0.861	1.138	0.8	**-0.250**	**-0.039**
**1**	100	-0.193	-0.315	0.122	0.942	1.644	0.6	**-0.250**	**-0.039**
**3**	25	-0.169	-0.260	0.091	2.141	2.731	0.78	**-0.215**	**-0.004**
**3**	50	-0.169	-0.261	0.092	3.118	3.792	0.82	**-0.215**	**-0.004**
**3**	100	-0.165	-0.264	0.099	4.249	5.239	0.81	**-0.215**	**-0.004**
**3**	200	-0.160	-0.269	0.109	5.76	7.209	0.8	**-0.215**	**-0.004**
**3**	500	-0.150	-0.278	0.128	8.265	10.53	0.78	**-0.214**	**-0.003**
**3**	1000	-0.142	-0.285	0.143	10.5	13.85	0.76	**-0.214**	**-0.003**

Supplementary Table 5 | The voltammetric data for complex **3** (10^{-3} M) in acetonitrile solution (0.1 M Bu_4NClO_4 as supporting electrolyte) at 298 K.

Compound	r (mV s^{-1})	$E_{p,a}$ (V) vs. Ag/Ag^+	$E_{p,c}$ (V) vs. Ag/Ag^+	ΔE_p (V)	$i_{p,a}$ (μA)	$i_{p,c}$ (μA)	$i_{p,a}/i_{p,c}$	**$E_{1/2}$ (V) vs. Ag/Ag^+**	**$E_{1/2}$ (V) vs. Fc/Fc^+**
**3**	10	+0.419	+0.341	0.078	3.114	3.003	1.04	**+0.380**	**+0.027**
**3**	25	-0.842<br>+0.418	-0.928<br>+0.341	0.086<br>0.077	3.552<br>4.812	5.374<br>4.375	0.66<br>1.1	**-0.885**<br>**+0.380**	**-1.238**<br>**+0.027**
**3**	50	-0.837<br>+0.418	-0.932<br>+0.341	0.095<br>0.077	5.424<br>6.636	7.130<br>5.954	0.76<br>1.12	**-0.885**<br>**+0.380**	**-1.238**<br>**+0.027**
**3**	100	-0.831<br>+0.415	-0.935<br>+0.343	0.104<br>0.072	7.596<br>8.835	8.426<br>8.435	0.9<br>1.05	**-0.883**<br>**+0.379**	**-1.236**<br>**+0.026**
**3**	200	-0.828	-0.941	0.113	10.59	12.23	0.87	**-0.885**	**-1.238**
Ferrocene	25	0.393	0.313	0.080				**0.353**	**0**

Supplementary Table 6 | Crystal data and structure refinement parameters for the X-ray structures of **2**, **3** and **5**.

Compound	**2**	**3**	**5**
Molecular formula	$C_{51}H_{91}Cl_{21}FeN_{14}O_6$	$C_{60}H_{78.564}As_2FeN_{12}O_{19.282}$	$C_{15}H_{32}FeN_{12}O_{13}Ca$
Formula wt. (g mol-1)	1796.67	1482.12	684.45
Temperature (K)	293(2)	296(2)	293(2)
Radiation (λ, Å)	0.71073	0.71073	0.71073
Crystal system	Triclinic	Orthorhombic	Triclinic
Space group	P-1	*Pbca*	P-1
a (Å)	13.1207(10)	21.0880(4)	9.4448(6)
b (Å)	15.5348(11)	21.0711(4)	12.0118(8)
c (Å)	22.3210(16)	29.6134(5)	12.3759(8)
α (°)	72.773(4)	90.00	87.085(4)
β (°)	77.360(4)	90.00	73.158(4)
γ (°)	70.252(4)	90.00	84.015(4)
Volume ($Å^3$)	4054.1(5)	13158.6(4)	1336.17(15)
Z	2	8	2
ρ_{calcd} (mg m^{-3})	1.472	1.496	1.701
μ (mm^{-1})	0.93	1.31	0.84
F(000)	1852	6151	712
crystal size (mm^3)	0.24 × 0.16 × 0.11	0.5 × 0.13 × 0.07	0.22 × 0.13 × 0.10
Theta range	1.5 to 26.5°	1.7 to 27.5°	1.9 to 26.1°
reflections collected	42541	139794	11544
independent reflections	16693 [R(int) = 0.057]	16323 [R(int) = 0.120]	5360 [R(int) = 0.107]
Compliteness	99.1%	99.9%	99.0%
goodness-of-fit on F^2	1.03	1.00	0.93
final R indices	$R1^a$ = 0.0925	$R1^a$ = 0.0529	$R1^a$ = 0.0745
[R > 2σ (I)]	$wR2^b$ = 0.2475	$wR2^b$ = 0.0961	$wR2^b$ = 0.1217
R indices (all data)	$R1^a$ = 0.1641	$R1^a$ = 0.1337	$R1^a$ = 0.1793
	$wR2^b$ = 0.2956	$wR2^b$ = 0.1230	$wR2^b$ = 0.1589
largest diff. peak and hole (e $Å^{-3}$)	1.32 and -0.99	1.02 and -0.51	0.66 and -0.68

Supplementary Table 7 | Optimized structure of complex anion of **3** (without counter cation and solvate water molecules) in the xyz format. The coordinates are in Å.

Fe	12.57696063354602	7.61047809708366	4.54295396641524
N	11.62532203742888	6.51327130433291	3.22758842860839
N	14.11560176779287	6.43000246888045	4.27571115327401
N	13.98193178648864	8.73575244351334	5.31617952643340
N	12.49757918386262	8.88626410185505	3.05949587321881
N	11.28117677430227	8.74845173106663	5.47111235337980
N	11.95992562208163	6.34531314230155	5.90566720554804
N	11.67266649949858	5.10339947137413	3.27594932196901
C	11.50471210097100	7.02447933908560	1.97779220605867
N	13.95756772831906	5.03093542791062	4.17232451829737
C	15.28935629401289	6.81820596552277	4.83230088928714
N	13.84860701462348	10.13993114733757	5.36848918389311
C	15.25889906791342	8.30326977850123	5.17060967846036
N	12.55156890096554	10.27825605011763	3.28768582783064
C	11.75140087935443	8.52794686917014	1.98535581433399
N	11.39323261896737	10.15562614727586	5.45045648963507
C	10.73560426044873	8.25022503225206	6.60800796517713
N	12.03649578507800	4.95048861525560	5.70065041424069
C	10.91526537274637	6.73850697981881	6.67486112782060
C	13.07035353086314	4.66204945279724	3.06954503377162
C	11.17714776526465	4.58564583578862	4.55111393804269
O	11.23356364924881	6.41228127283119	0.92856470229049
C	13.40922083832590	4.51248835321322	5.44584509630372
O	16.29059624602088	6.11618515218371	5.06350823052117
C	13.77479343359169	10.66841409945097	3.98728843596741
C	12.65757560342240	10.54645838769751	6.11445136562969
O	16.30423325594315	8.96943890958234	5.28149863496976
C	11.37192273071627	10.68206461058882	4.08593118334951
O	11.29758860477058	9.26848872763096	1.09412650476392
O	10.15659519976436	8.87936138041998	7.51229531740737
O	10.16605397712194	6.02215317491060	7.36387539603057
H	13.07247210575719	3.56552268810691	2.99394612362937
H	13.44266293682172	5.10014899806301	2.13728278763102
H	11.15559083317052	3.48902916957285	4.47910757518092
H	10.16661041002697	4.96707535687139	4.73379017667857
H	13.40227773996634	3.41465809786119	5.38966942382172
H	14.05767271162069	4.83994403476339	6.26613459908480
H	13.78988222867527	11.76595230944547	4.04437119272254
H	14.64702049652446	10.31165864390031	3.42805819262825
H	12.67346622428202	11.64261909982727	6.19497441137463
H	12.68038678790311	10.09948629561404	7.11406555259225
H	11.36707269127770	11.77901780633139	4.15236103399402
H	10.46772567051835	10.33417402989198	3.57397314773250

Supplementary Table 8 | Calculated additional Mössbauer parameters for **3**: electronic density on Fe nucleus (ρ) and electric field gradient (V) principal components with orientation. All quantities are in a.u.$^{-3}$. OPT denotes calculation using DFT optimized structure.

Complex **3**	V_{xx}	V_{yy}	V_{zz}	**ρ**
	0.6623	0.8132	-1.4755	14764.152946867
Orientation				
X	-0.7397743	0.6728201	-0.0068678	
Y	-0.0379753	-0.0315594	0.9987802	
Z	0.6717826	0.7391327	0.0488974	
OPT				
	0.7672	0.7752	-1.5423	14764.148581815
Orientation				
X	0.7848503	0.6196505	-0.0065718	
Y	0.0326826	-0.0519814	-0.9981131	
Z	-0.6188229	0.7831546	-0.0610494	

Supplementary Table 9 | List of complexes used to calibrate B3LYP/TZVP method for the Mössbauer calculations. Exp denotes isomer shift experimental numbers (in mm/s), RHO – electron density on the Fe nucleus (in a.u.$^{-3}$). The ligands in the complexes are defined in Supplementary references[2-4].

	exp	RHO	RHO-14763
FeCO5S0 – $Fe(CO)_5$	0	14764,6422	1,642195268
FeIICl42minS2 – $[FeCl_4]^{2-}$	0,9	14761,89429	-1,105706135
FeIICN64minS0 – $[Fe(CN)_6]^{4-}$	-0,02	14764,1197	1,11969666
FeIIF64minS2 – $[FeF_6]^{4-}$	1,34	14760,53652	-2,463483171
FeIIIAzPlusS0p5 – $[FeAz]^{+}$	0,29	14763,44985	0,449845367
FeIIICl4minS2p5 – $[FeCl_4]^{-}$	0,19	14763,58943	0,589425222
FeIIICN63minS0p5 – $[Fe(CN)_6]^{3-}$	-0,13	14764,39093	1,390925797
FeIIIF63minS2p5 – $[FeF_6]^{3-}$	0,48	14762,70495	-0,295047988
FeIIIH2O63plusS2p5 – $[Fe(H_2O)_6]^{3+}$	0,51	14762,76603	-0,233966526
FeIIIMAC2minS1p5 – $[Fe(MAC)]^{2-}$	0,15	14763,79716	0,797162875
FeIIIOEPPYplusS0p5 – $[Fe(OEPPY)]^{+}$	0,2	14763,58837	0,588374388
FeIIIPorMinS2p5 – $[Fe(Por(O2))]^{-}$	0,67	14762,39268	-0,607319513
FeIIIPorOAcS2p5 – [Fe(Por(OAc))]	0,4	14763,23073	0,230730075
FeIIPorOACminS2 – $[Fe(Por(OAc))]^{-}$	1,05	14761,80596	-1,194035088
FeIISR3minS2 – $[Fe(SR_3)]^{-}$	0,56	14763,47473	0,474731754
FeIVMACminS2 – $[Fe(MAC)]^{-}$	-0,02	14764,47098	1,470983361
FeIVTMCO2plusS1 – $[Fe(TMCO)]^{2+}$	0,08	14764,01418	1,014181498
FeNO6plusS0 – $[\{FeNO\}^{6}]^{+}$	0,04	14764,16358	1,163579948
FeNO7S0p5 – $[\{FeNO\}^{7}]$	0,33	14763,63752	0,63752386
FePH3S0 – $[Fe(PH_3)]$	0,34	14763,36037	0,360367575
FeSMES0 – [Fe(SME)]	0,44	14763,15817	0,158168215
FeVIO42minS1 – $[FeO_4]^{2-}$	-0,87	14767,12488	4,124876889

Supplementary Table 10 | Calculated energetics for different spin states of the complex 3 in X-Ray geometry. The total energies are given in Hartree. The energy differences are given in kcal/mol.

	Total Energy	Energy Difference
S=0	-2852.04157605	22
S=1	-2852.07696142	0
S=2	-2852.03275811	28

Supplementary Table 11 | Population analysis based on B3LYP/TZVP electron density of complex **3** in BP86/TZVP optimized geometry. All numbers are in atomic units.

Loewdin	Atomic Charge	Spin Population
Fe	0.606984	1.803976
Mulliken		
Fe	0.172992	1.902444

Supplementary Methods

Computational details.

All DFT calculations were carried out using the ORCA quantum chemistry program package[5,6]. The starting geometry was derived from the crystallographic data without taking counter cation and solvate molecules into modelling. Full optimization was carried out using the BP86[7,8] functional. The resolution-of-the-identity (RI) approximation[9,10] and a triple-ζ basis set (TZVP[11]) together with auxiliary basis set (TZV/J[12] in ORCA notation) were applied. The conductor-like screening model (COSMO)[13] was applied to account for solvent effects with the dielectric constant of water. Relativity was added through the zero-order regular approximation (ZORA)[14]. The calculations utilized the atom-pairwise dispersion correction with the Becke-Johnson damping scheme (D3BJ)[15,16]. The requested total energies convergence was $5 \cdot 10^{-6}$ hartree. Geometry optimizations were performed with the convergence threshold of 10^{-4} for the RMS gradient and $3 \cdot 10^{-4}$ for the maximum component of the gradient (in hartree/bohr units). The stationary optimized geometries were then verified by performing molecular Hessian calculations according to the method outlined in[17].

The isomer shift and quadrupole splitting were then obtained from single point B3LYP[18,19]/TZVP calculations on the optimized structures as well as on the crystallographycally determined geometries. To obtain reliable Mössbauer parameters from DFT calculations an additional flexibility of the basis set in the nuclear region and higher integration accuracy are required. For this purpose the ORCA "core properties" CP(PPP) basis set was used with the radial integration accuracy parameter increased to 9.0 for iron centre.

Nuclear quadruple coupling constants, e^2qQ/h, were calculated from the electric field gradients V_{ii} according to the equation $e^2qQ/h = const*V_{ii}*Q$, where Q is the nuclear quadrupole

moment (Q(Fe) = 0.16 barn). The factor const = 234.96 serves to convert e^2qQ/h from the atomic to MHz units. The deviation of the nuclear quadrupole tensor from axial symmetry is given by the asymmetry parameter $\eta = (V_{xx} - V_{yy})/V_{zz}$ in a coordinate system where $|V_{zz}|>|V_{yy}|>|V_{xx}|$.

For the purpose of analysis, the unrestricted Kohn-Sham orbitals were localized according to Pipek-Mezey localization procedure[20]. Orbitals, densities and structures were visualized with the Chimera program[21].

Supplementary References

1. Gütlich, P., Bill, E. & Trautwein, A. X. *Mössbauer Spectroscopy and Transition Metal Chemistry: Fundamentals and Applications*. (Springer-Verlag: Berlin, 2011).

2. Sinnecker, S., Slep, L. D., Bill, E. & Neese, F. Performance of nonrelativistic and quasi-relativistic hybrid DFT for the prediction of electric and magnetic hyperfine parameters in Fe-57 Mossbauer spectra. *Inorganic Chemistry* **44**, 2245-2254 (2005).

3. Neese, F. Prediction and interpretation of the Fe-57 isomer shift in Mossbauer spectra by density functional theory. *Inorganica Chimica Acta* **337**, 181-192 (2002).

4. Romelt, M., Ye, S. F. & Neese, F. Calibration of Modern Density Functional Theory Methods for the Prediction of Fe-57 Mossbauer Isomer Shifts: Meta-GGA and Double-Hybrid Functionals. *Inorganic Chemistry* **48**, 784-785 (2009).

5. ORCA, version 3.0 (MPI CEC, Mülheim a.d. Ruhr, Germany, 2015).

6. Neese, F. The ORCA program system. *Wiley Interdiscip. Rev.-Comput. Mol. Sci.* **2**, 73-78 (2012).

7. Perdew, J. P. Density-functional approximation for the correlation energy of the inhomogeneous electron gas. *Phys Rev B* **33**, 8822-8824 (1986).

8. Becke, A. D. Density Functional Calculations of Molecular-Bond Energies. *J Chem Phys* **84**, 4524-4529 (1986).

9. Dunlap, B. I., Connolly, J. W. D. & Sabin, J. R. Some Approximations in Applications of X-Alpha Theory. *J Chem Phys* **71**, 3396-3402 (1979).

10. Baerends, E. J., Ellis, D. E. & Ros, P. Self-consistent molecular Hartree-Fock-Slater calculations - I. The computational procedure. *Chemical Physics* **2**, 41-51 (1973).

11. Schafer, A., Huber, C. & Ahlrichs, R. Fully Optimized Contracted Gaussian-Basis Sets of Triple Zeta Valence Quality for Atoms Li to Kr. *J Chem Phys* **100**, 5829-5835 (1994).

12. Eichkorn, K., Weigend, F., Treutler, O. & Ahlrichs, R. Auxiliary Basis Sets for Main Row Atoms and Transition Metals and Their Use to Approximate Coulomb Potentials. *Theor. Chem. Acc.* **97**, 119-124 (1997).

13. Klamt, A. & Schuurmann, G. COSMO: a new approach to dielectric screening in solvents with explicit expressions for the screening energy and its gradient. *Journal of the Chemical Society Perkin Transactions 2*, 799-805 (1993).

14. van Wüllen, C. Molecular density functional calculations in the regular relativistic approximation: Method, application to coinage metal diatomics, hydrides, fluorides and chlorides, and comparison with first-order relativistic calculations. *J. Chem. Phys.* **109**, 392 (1998).

15. Grimme, S., Antony, J., Ehrlich, S. & Krieg, H. A consistent and accurate ab initio parametrization of density functional dispersion correction (DFT-D) for the 94 elements H-Pu. *J Chem Phys* **132**, 154104-154119 (2010).

16. Grimme, S., Ehrlich, S. & Goerigk, L. Effect of the Damping Function in Dispersion Corrected Density Functional Theory. *Journal of Computational Chemistry* **32**, 1456-1465 (2011).

17. Bykov, D. *et al.* Efficient implementation of the analytic second derivatives of Hartree-Fock and hybrid DFT energies: a detailed analysis of different approximations. *Mol Phys* **113**, 1961-1977 (2015).

18. Lee, C., Yang, W. & Parr, R. G. Development of the Colle-Salvetti correlation-energy formula into a functional of the electron density. *Phys. Rev. B.* **37**, 785-789, doi:10.1103/PhysRevB.37.785 (1988).

19. Becke, A. D. Density-functional thermochemistry. III. The role of exact exchange. *J. Chem. Phys.* **98**, 5648-5652, doi:10.1063/1.464913 (1993).

20. Pipek, J. & Mezey, P. G. A Fast Intrinsic Localization Procedure Applicable for Abinitio and Semiempirical Linear Combination of Atomic Orbital Wave-Functions. *J Chem Phys* **90**, 4916-4926, doi:10.1063/1.456588 (1989).

21. Pettersen, E. F. *et al.* UCSF chimera - A visualization system for exploratory research and analysis. *Journal of computational chemistry* **25**, 1605-1612, doi:10.1002/Jcc.20084 (2004).
